# Transport and senescence

**DOI:** 10.18632/oncoscience.191

**Published:** 2015-08-12

**Authors:** David Bernard, Clotilde Wiel

**Affiliations:** Inserm U1052, Centre de Recherche en Cancérologie de Lyon, CNRS 5286, Centre Léon Bérard, Université de Lyon, Lyon, France

**Keywords:** senescence, ABCC3

In response to various cellular stresses, most of the cells can enter cellular senescence. Senescence is a proliferation arrest characterized by several phenotypic and molecular changes such as an enlarged and flat morphology and an increased secretion of soluble cytokines, chemokines and metalloproteases known as SASP (Senescence-Associated Secretory Phenotype). This response appear to play important roles in physiological and pathophysiological conditions, notably when cells undergo oncogenic stress. Indeed, oncogenic stress results in Oncogene-Induced Senescence (OIS) which has to be overcome to allow tumor formation.

Most studies investigating mechanisms underlying OIS mechanisms were performed on fibroblasts. However, carcinoma, type of cancer whose cellular origin is the epithelial compartment, represents most of solid cancers. Moreover, it clearly emerges now that responses to stress are in part cell type dependent, revealing different, but non-exclusive, mechanisms regulating OIS in fibroblasts, epithelial cells or melanocytes [1, 2]. These observations underline that OIS investigation in epithelial cells might reflect more closely events happening *in vivo*.

To uncover new mechanisms regulating OIS in epithelial cells, we performed in immortalized mammary epithelial cells a loss-of-function genetic screen using a shRNA library covering the annotated genome. In response to oncogenic activation of the MAPK pathway, these cells enter senescence. Cells recovering from this proliferation arrest were amplified and their integrated shRNA isolated and identified.

Surprisingly this method led us to identify several genes whose main function is molecule transport. Amongst them we characterized KCNA1, a voltage-dependent potassium channel. Oncogenic activation results in KCNA1 relocalization to the membrane and change in membrane potential. We speculated that regulation of the extracellular potassium concentration mediated notably by KCNA1, and the resulting state of membrane potential, is a key event in cellular response to oncogenic stress [3]. Interestingly, KCNA1 was not the only channel identified in our screen as two calcium channels, ITPR2 (IP R2) and MCU, were also involved in regulating the outcome of the cells in response to oncogenic stress. Calcium exchange between the endoplasmic reticulum, through IP_3_R2, and the mitochondria, via MCU, proved to be essential for establishing the stress-induced growth arrest [4]. These 2 studies highlight how intra- and extra-cellular ion transport play a major role in establishing senescence.

ABCC3 gene (also named MRP3) also emerged from our screen. Because this gene encodes a protein known to transport molecules outside the cells, it held up our attention and we decided to further investigate its function in senescence. Down-regulating ABCC3 protein expression allowed epithelial cells to escape OIS. To confirm these *in vitro* observations, we used a model of a 2-step carcinogenesis (DMBA-TPA protocol) where OIS bypass in epithelial cells is necessary for carcinoma development. Tumor burden was increased in ABCC3 knock-out mice, as carcinoma developed earlier and their incidence was higher than in control littermates. Importantly, DMBA-TPA-treated skin from ABCC3 KO mice were marked by weaker staining for senescence markers and by a higher proliferation index. In accordance with these results, microarray analyses revealed a loss of ABCC3 expression in skin squamous cell carcinoma and in skin basal carcinoma. To understand the mechanism giving ABCC3 these tumor suppressive properties, we investigated its transport function. Whereas overexpression of ABCC3 sensitized cells to OIS, overexpression of a mutant form of ABCC3 whose transport function is altered was unable to sensitize cells to OIS [5]. Altogether, these observations led us to speculate that ABCC3 transporter excretes outside the cells macromolecules necessary for OIS establishment. Identifying ABCC3 substrates in response to oncogenic stress will allow to deeper understand how secretion of molecules participates in senescence response. Interestingly ABCC1 (MRP1) overexpression resulted in a growth arrest that rapidly turned into cell death (unpublished data). This observation could be explained by either the nature and/or the concentration of MRP1 substrates that could be toxic for the cells. Nevertheless, these results underline that molecule export is hardly insignificant for cellular outcome and suggest that several transporters from the ATP-binding cassette superfamily could play a previously unknown role in senescence response.

**Figure 1:**
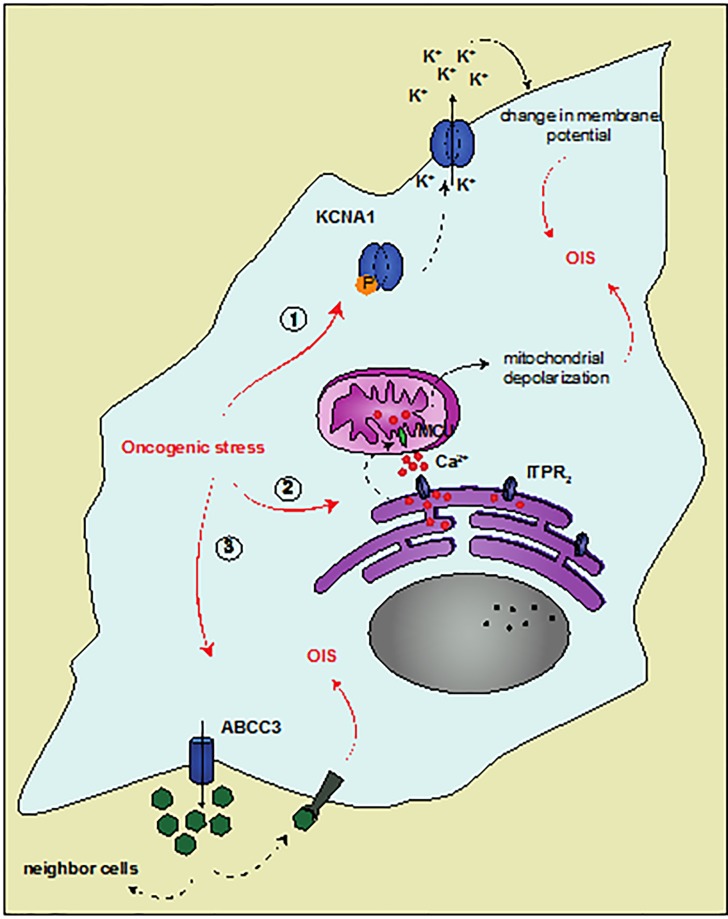
New forms of transport in senescent cells 1. Oncogenic stress induces KCNA1 calcium relocalization to the membrane. Potassium transport outside the cells results in a change in membrane potential that might contribute to senescence. 2. During oncogenic stress, a transfer of calcium between the endoplasmic reticulum and the mitochondria is observed, through the ITPR2 and MCU channels. The following calcium overload results in dysfunctional mitochondria, contributing to the senescence response. 3. ABCC3 takes part in the outcome of the cells by exporting macro-molecules essential for OIS establishment in a paracrine or autocrine manner.

A lot of evidence point out communication systems as a hot spot within senescent cells. The SASP, characterized by the secretion of soluble factors such as IL-6 and IL-8 interleukins, has been extensively studied in the last few years and is recognized as a major player in this system [6]. More recently, it was shown that senescent cells also communicate with neighbor cells via the creation of cytoplasmic bridges and direct transfer of proteins [7]. In these studies, cell communication is a key point to immune cells attraction and activation, leading to the elimination of senescent cells. Our results also show that transport functions in senescent cells can also take the form of transport of ions or macromolecules, pointing out new possible ways for senescent cells to propagate stress signals in an intracrine/autocrine or paracrine manner.
